# Results of a Retrospective Fracture Register of Distal Radius Fractures Built Up Using PROM

**DOI:** 10.3389/fsurg.2022.854828

**Published:** 2022-04-01

**Authors:** Johannes Rammensee, Francesca von Matthey, Peter Biberthaler, Helen Abel

**Affiliations:** Department of Trauma Surgery, Klinikum Rechts Der Isar, Technische Universität München, Munich, Germany

**Keywords:** register study, patient-related outcome measurements, distal radius fracture, PROM, fracture register, Munich Wrist Questionnaire

## Abstract

**Introduction:**

Although distal radius fractures (DRFs) are the most common fractures of the human body, the best treatment for every fracture type is still debatable. However, randomized controlled trials are difficult to perform. The quality of care can be determined primarily in the context of health care research using register studies. Registers enable standardized documentation of clinical observations over time. So far, no German register studies concerning DRFs exist, and therefore, the aim of this study was to develop a register with the help of patient-reported outcome measurements (PROM).

**Patients and Methods:**

All patients treated surgically at our hospital with a DRF between 2006 and 2016 were enrolled. Patient data such as epidemiological data, treatment, complications, insurance status, etc. were collected and the register was built up as an in-house fracture register with the help of PROM. The Munich Wrist Questionnaire (MWQ) was used as a PROM tool.

**Results:**

Of all 1,796 patients, 339 (19%) with a complete data set could be enrolled, 96 of the patients were male (28%), 243 were female (72%). Thirty-two percent were type A (*n* = 110), 9% (*n* = 31) were type B, and 58% (*n* = 198) were type C fractures. The average follow-up was 66 ± 31 months. Complications occurred in 25 cases (7%). The average postoperative function measured with the MWQ was 91 ± 11%. Patients suffering from a DRF type A had the best outcome. It was significantly better than the outcome of patients with a DRF type C (95 ± 7 vs. 89 ± 13%, *p* < 0.05 MWUT) and significantly better compared to the results from the whole fracture register (95 ± 7 vs. 91 ± 11%, *p* < 0.05 MWUT). Type B fractures had a better outcome than type C fractures (92 ± 11%).

**Conclusions:**

Retrospective register studies created with the help of PROM have numerous advantages. Data collection is fast, easy and cost-effective and a huge amount of data can be achieved from numerous patients and the observation period after surgery is quite long. The drop-out rate might be high, but patients enrolled are a representative sample compared to the current literature. This is a valuable tool for monitoring of clinical treatment quality.

## Introduction

Distal radius fractures (DRFs) are the most common fractures of the human body all over the world (1). This fracture type has two incidence peaks: young people, mostly young men and elderly women. While this fracture in young patients is mostly associated with a high-energy trauma, in older patients a low-energy trauma like fall from a standing position is common. This is mostly due to underlying osteoporosis or osteopenia (1–3).

Treatment options are discussed widely in the literature. Within the recent decades, the trend is more to the operative treatment and several studies could show a good outcome after surgery even for the elderly patient (4–7). Nevertheless, the optimal treatment options for different fracture types and patient categories are still debated (5, 8, 9). Discussing conservative and operative treatment options, arguments for a conservative treatment are often based on the age of the patients whereas supporters of the operative treatment argue with the need for a good wrist function and fast recovery especially in the elderly.

As there is a certain heterogeneity concerning therapeutic procedures, safety, and effectiveness of the various therapy procedures should be constantly reevaluated. In the question of fracture treatment, however, randomized controlled trials (RCTs) are difficult to use here, especially when comparing operative with conservative fracture treatment. Whether a treatment strategy is successful under everyday clinical conditions, i.e., the quality of care, can be determined primarily in the context of health care research using registry studies. Registers enable standardized documentation of clinical observations over time.

Only a few register studies exist and to our knowledge, so far no register study about the distal radius fracture dealing not only with the objective function but also with the subjective outcome of the patients evaluated by a self-assessment score exists (10, 11).

The aim of this study was to develop a primarily clinical-internal fracture register of distal radius fractures for the internal quality assurance of the treatment. This includes data concerning epidemiology, fracture classification, injury characteristics and current treatment regimens in our in-house population within the last 10 years for internal quality within the context of a large in-house register study. The MWQ not only collects objective data on the wrist function, but also measures subjective patient satisfaction as part of a Patient Reported Outcome Measure (PROM) (12).

As part of our study, we were able to establish a fracture register that covers the treatment of all operatively treated distal radius fractures within 10 years. In addition to collecting retrospective observation data, we were able to collect postoperative outcome using the MWQ.

## Patients and Methods

### Study Population and Data Collection

This retrospective cohort study was approved by the local ethics committee (409/15s) and all control individuals as well as patients gave their written informed consent prior to participation.

All non-pathological distal radius fractures, which have been treated in our clinic in the time period between 2006 and the 2016 were enrolled. Exclusion criteria were no signed letter of acceptance for the study and age under 18 years. The register was built up as an in-house fracture register for internal quality control with the help of patient reported outcome measurements (PROM).

### Variables

Epidemiological data from the patients (age and sex), insurance status (statutory insurance, private insurance, and trade association insurance), fracture data (fracture type, side, dominant vs. non-dominant wrist), treatment data (surgical procedure, implant type), complications, and PROM (MWQ results) were collected. As the AO classification is well-established internationally, fractures were classified according to it.

PROM was used to measure our main outcome variable, the function. We used the Munich Wrist Questionnaire (MWQ), a validated self-assessment questionnaire, published in 2016 by Beirer et al. (12). The MWQ-Score is measured in percent of the maximum number of points. Sent back MWQs were evaluated and converted into a percent value. We sent PROM to every surgically treated Patient with a distal radius fracture in the given period with a request to participate in our study. Received MWQs were checked on missing data or other exclusion criteria. The remaining patients were included in our fracture register.

### Statistics

Nominal variables are presented as proportions of all registered fractures, excluding any missing data. The surveyed Scale variables are presented as Mean ± Standard Deviation (SD).

To evaluate the outcome of the MWQ the points achieved in the questionnaire were converted into percent of the maximum value.

The additional statistical analysis was carried out using the SigmaPlot statistical program by Systat Software GmbH.

As most data was non-normally distributed, we used the Man–Whitney *U-*Test (MWUT) to compare different values.

## Results

### Epidemiological Data

**Figure 1:**
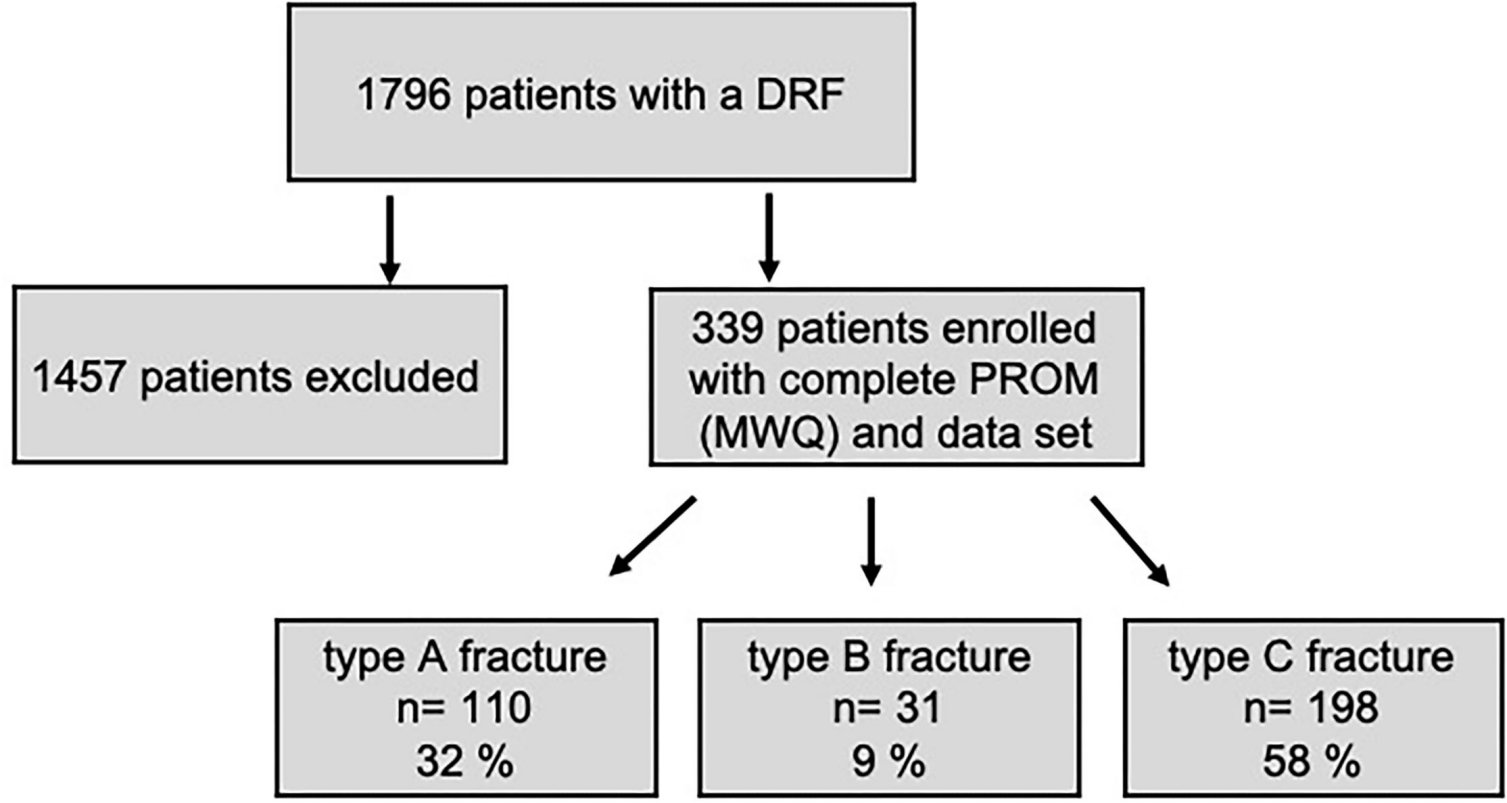
Study design with drop-out rate and patient enrollment. The subdivision into different fracture types is depicted as well.

Of all 1,796 patients who were treated for a distal radius fracture in our trauma center from 2006 to 2016, 339 (19%) patients with a complete data set could be included in our fracture register (Figure 1).

**Figure 2:**
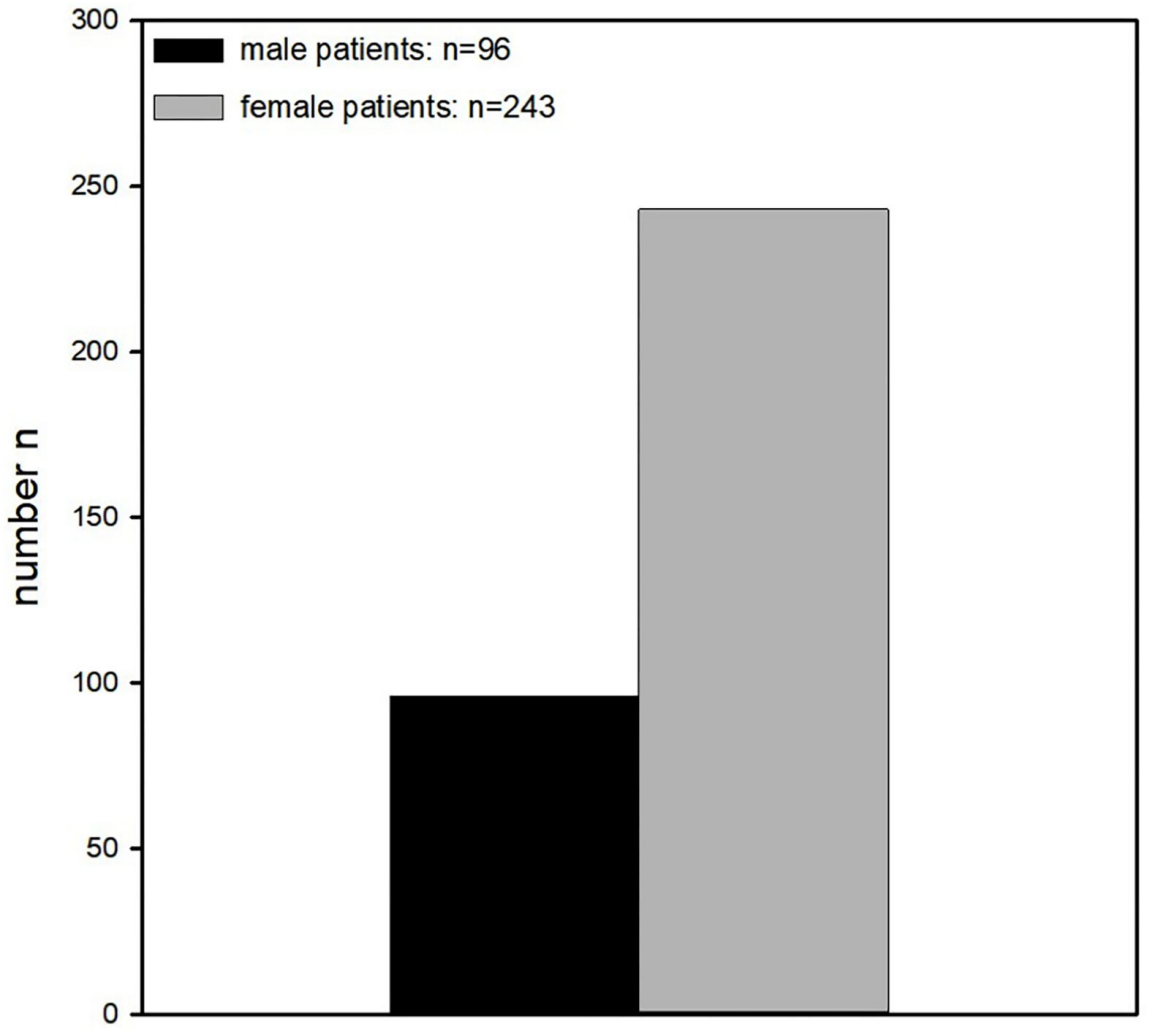
Gender distribution. Significantly more women than men were enrolled in this study (96 vs. 243).

Ninety-six of the patients were male (28%), 243 were female (72%) (Figure 2).

**Figure 3:**
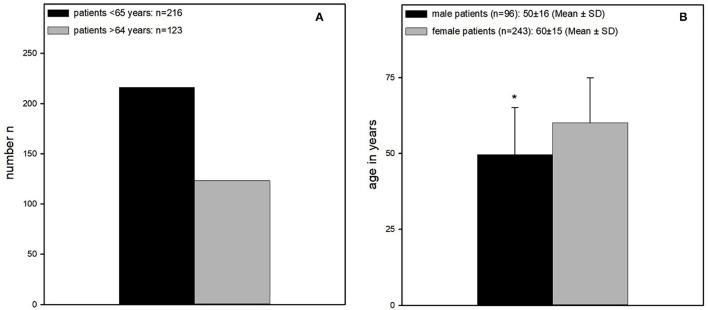
**(A)** Age distribution. Significantly more patients younger than 65 years could be enrolled (patients older than 64 years *n* = 123 vs. patients younger than 65 years, *n* = 216). **(B)** Mean age of male and female patients. Female patients were significantly older than male patients (60 ± 15 years vs. 50 ± 16 years).

The average age of the patients was 57 ± 16 years (Mean ± SD). Two hundred and sixteen of those were younger than 65 years old (64%) and 123 older than 64 years (36%). Female patients were significantly older than the male (60 ± 15 vs. 50 ± 16) (Figure 3).

The average follow-up of the 339 patients was 66 ± 31 months (Mean ± SD).

### Insurance Status

We differentiated between general insurance, private insurance and workers insurance. Overall, the majority of patients were generally insured with a total of 216 patients (64%), 41(12%) had a worker's insurance and 84 (25%) were privately insured or self-paying.

### Fracture Classification and Characteristics

Of all 339 affected wrists, 147 fractures affected the right and 192 fractures the left side, thus the left side was affected significantly more often than the right side. In 19 cases, bilateral fractures occurred.

Of all 339 patients, 145 patients had a fracture of the dominant wrist (43%) and 194 had a fracture of the non-dominant wrist (57%).

**Figure 4:**
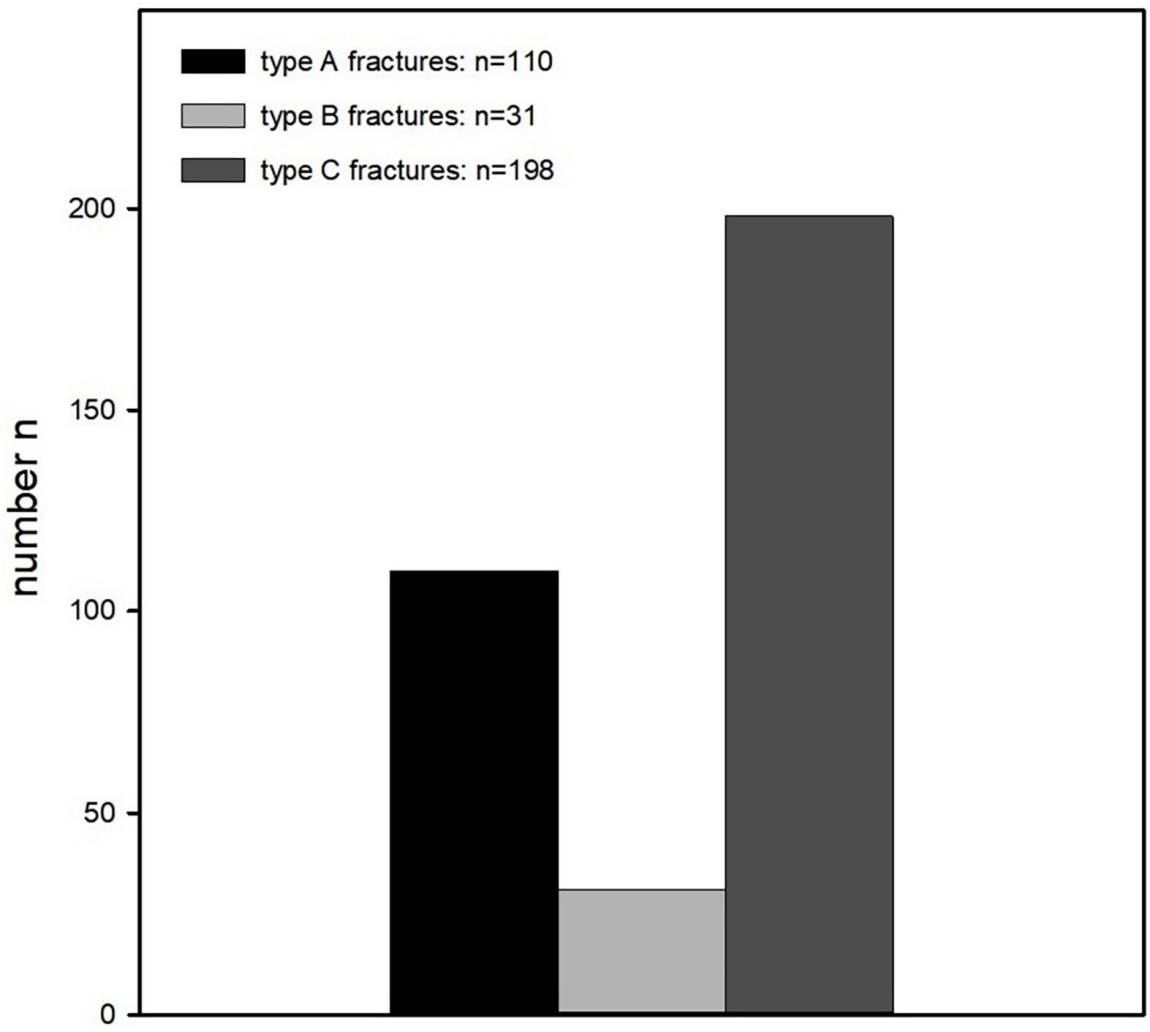
Classification of the fractures according to the AO classification. Fractures were classified as follows: type A fractures *n* = 110, type B fractures *n* = 31, and type C fractures *n* = 198.

Of all fractures analyzed by PROM, 32% were type A fractures (*n* = 110), 9% (*n* = 31) were type B fractures, and 58% (*n* = 198) were type C fractures (Figure 4).

### Treatment Data (Surgical Procedure, Implant Type)

Of the 339 distal radius fractures treated by surgery, 282 cases (83%) were subjected to plate osteosynthesis, in 7 cases (2%) screws and in 50 (15%) cases combined systems like k-wire and plate- or dual plate osteosynthesis. Fifty-three cases (16%) were primarily treated with an fixateur externe.

Most plates used for plate osteosynthesis were Aptus plates from Medartis (224; 79%), second most used plates were from Synthes (45; 16%).

Overall, the distal radius fractures have been treated by 57 different surgeons within the past 10 years.

The operation time varied from 16 to 322 min and averaged 81 ± 43 min. In 102 operations times were not documented.

### Complications

**Figure 5:**
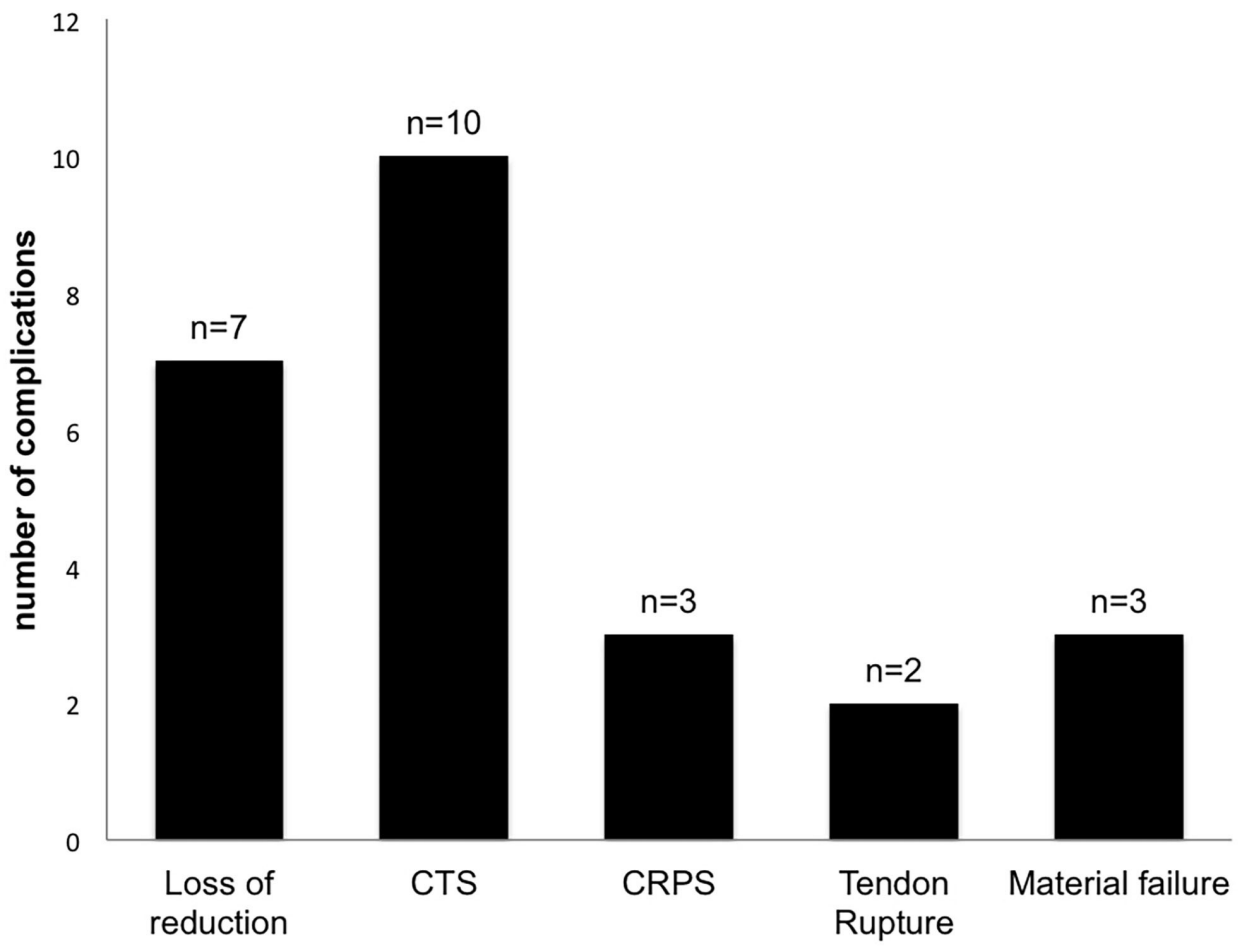
Complications. Complications were documented in 25 cases (7%), containing loss of reduction (*n* = 7), carpal tunnel syndrome (*n* = 10), complex regional pain syndrome (CRPS) (*n* = 3), material failure (*n* = 3), and tendon rupture (*n* = 2).

Complications were documented in 25 cases (7%), containing loss of reduction (*n* = 7), carpal tunnel syndrome (*n* = 10), Complex regional pain syndrome (CRPS) (*n* = 3), material failure (*n* = 3), and tendon rupture (*n* = 2) (Figure 5).

Surgical revisions were performed in 10 cases (0.4%).

Broken Screws or k-wires were counted as a material failure.

### PROM MWQ

**Figure 6:**
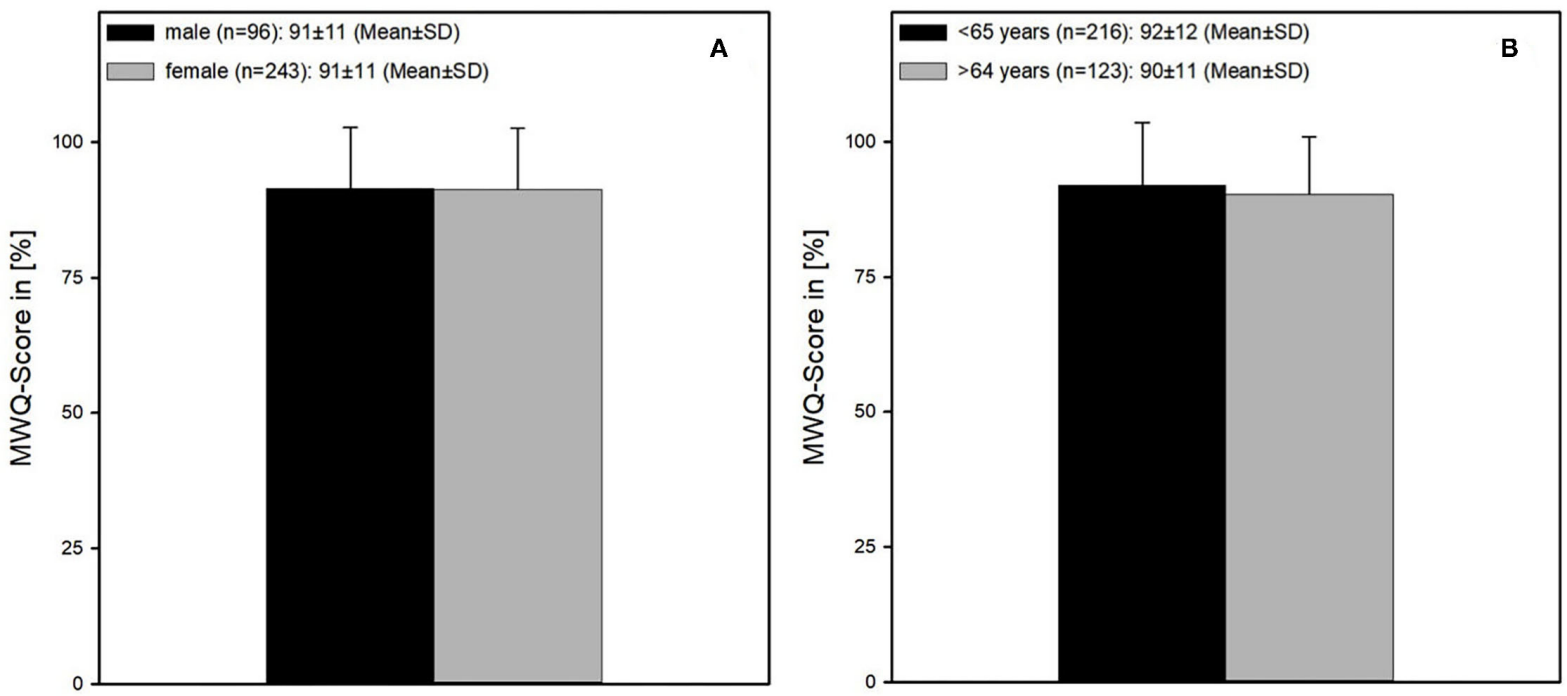
Wrist function according to the MWQ. **(A)** Male and female patients showed no difference concerning the wrist function (both 91 ± 11% MWQ-Score). **(B)** Age also had no influence on function regarding the patients older and younger than 65 years (age <65 and >64 years = 92 ± 12 vs. 90 ± 11).

The average postoperative function measured with the MWQ was 91 ± 11%, while both male and female patients had similar values (each 91 ± 11%). No significant difference was found (Figure 6A).

Patients younger than 65 years had a slightly better outcome compared to patients older than 64 years (92 ± 12 vs. 90 ± 11%) (Figure 6B).

**Figure 7:**
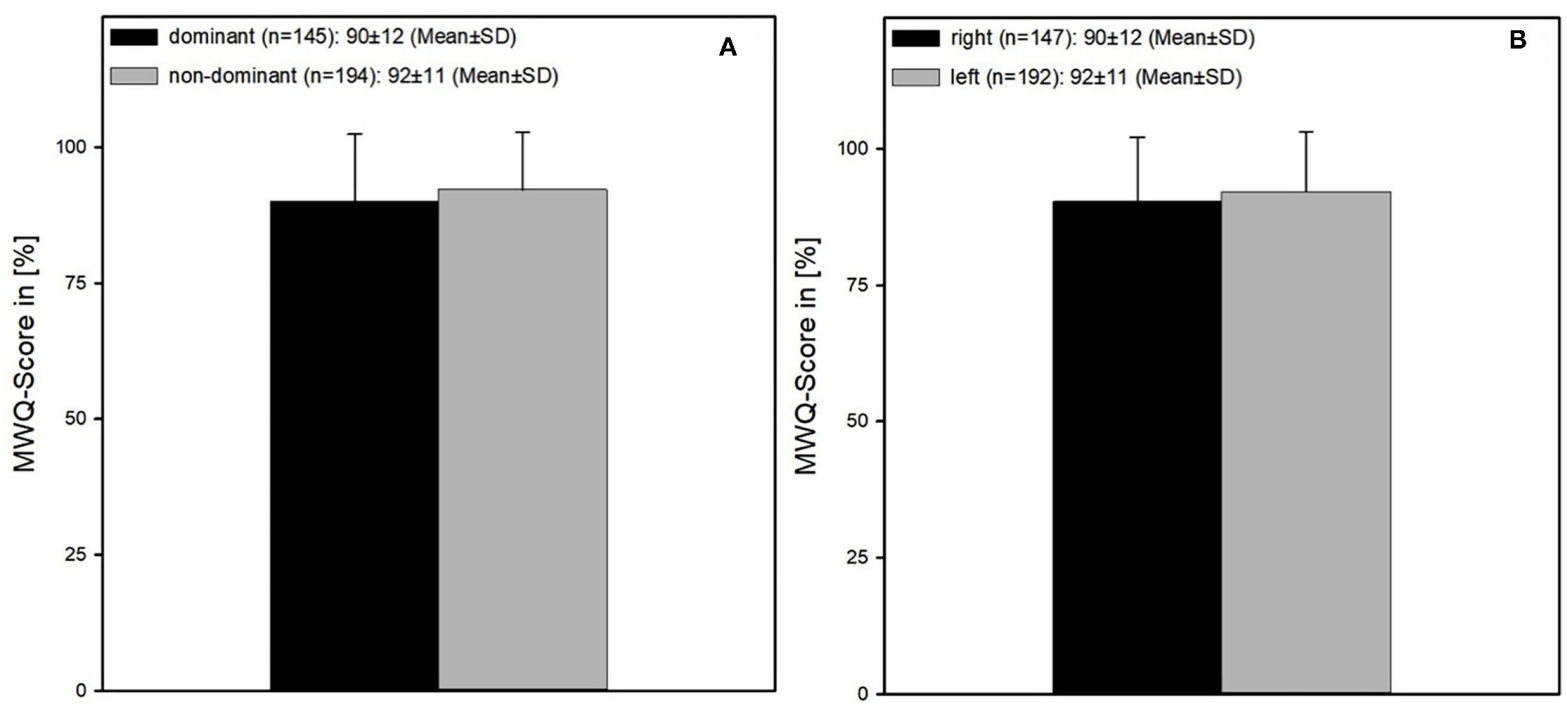
**(A)** Comparing the function after a fracture of the dominant and non-dominant wrist, respectively. The outcome of the dominant wrists was worse compared to the non-dominant wrists (90 ± 12 vs. 92 ± 11%, *p* < 0.072 MWUT). **(B)** Comparing the function after a fracture of the left and right wrist, respectively. Results after a fracture of the right wrist were worse compared to the fractures of the left wrist (90 ± 12 vs. 92 ± 11%, *p* < 0.064 MWUT).

Patients with a fracture of the right wrist had a worse outcome compared to patients with a fracture of the left wrist (90 ± 12 vs. 92 ± 11%, *p* < 0.064 MWUT) (Figure 7B).

This comes along with the outcome of the analysis of the dominant vs. non-dominant wrist as patients with a fracture of the dominant wrist also had a worse outcome compared with those suffering from a fracture of the non-dominant wrist (90 ± 12 vs. 92 ± 11%, *p* < 0.072 MWUT) (Figure 7A).

**Figure 8:**
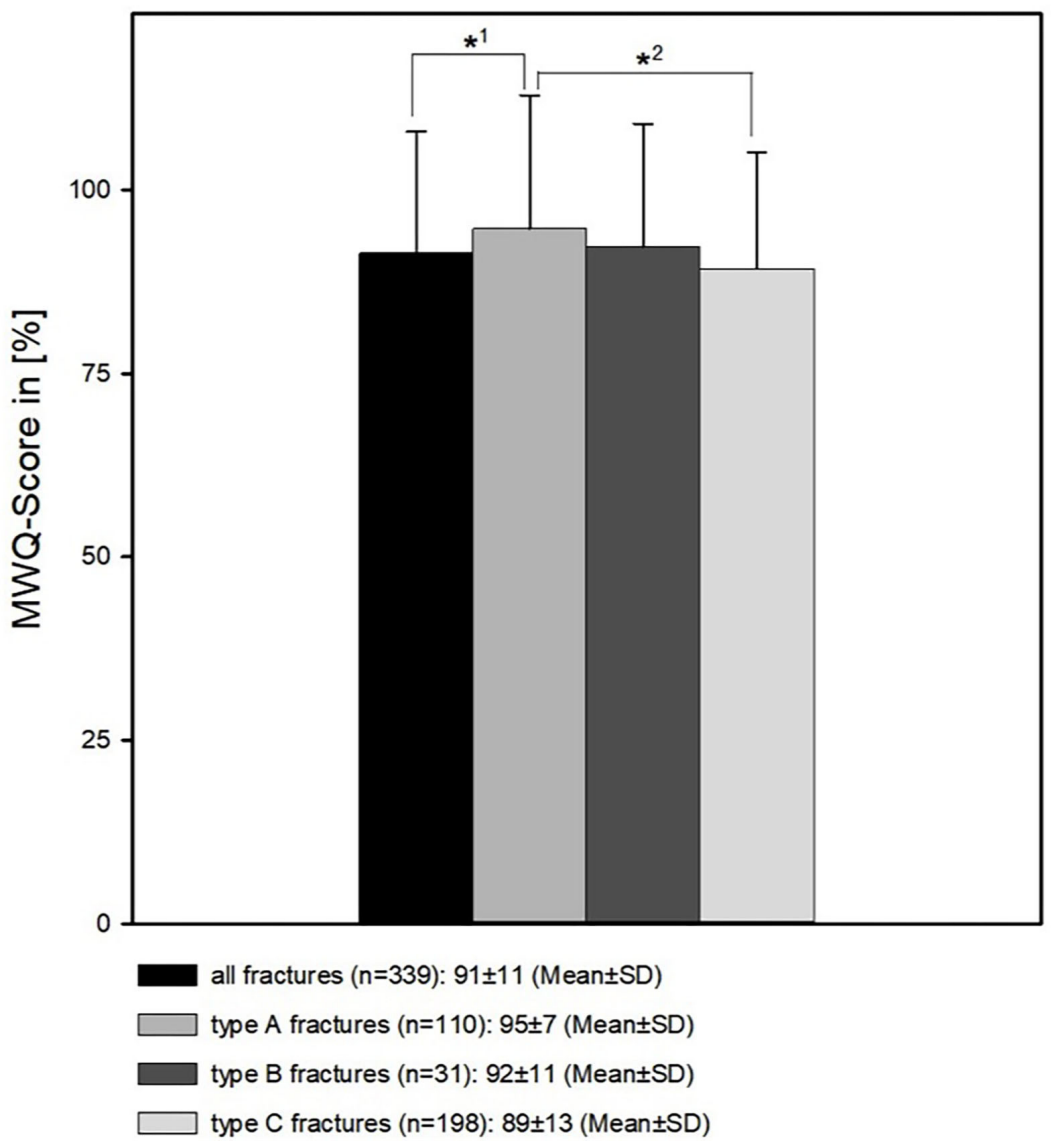
Patients' wrist function in respect to the different fracture types. *2 The function of type A fractures is significantly better than type C fractures (95 ± 7% vs. 89 ± 13%, *p* < 0.05 MWUT). *1 The function of type A fractures is significantly better than the results from the whole fracture register (95 ± 7% vs. 91 ± 11%, *p* < 0.05 MWUT).

Regarding the different fracture types, patients suffering from a DRF type A had the best outcome. It was significantly better than the outcome of patients with a DRF type C (95 ± 7% vs. 89 ± 13%, *p* < 0.05 MWUT) and significantly better compared to the results from the whole fracture register (95 ± 7 vs. 91 ± 11%, p <0.05 MWUT). Type B fractures had a better outcome than type fractures (92 ± 11%) (Figure 8).

**Figure 9:**
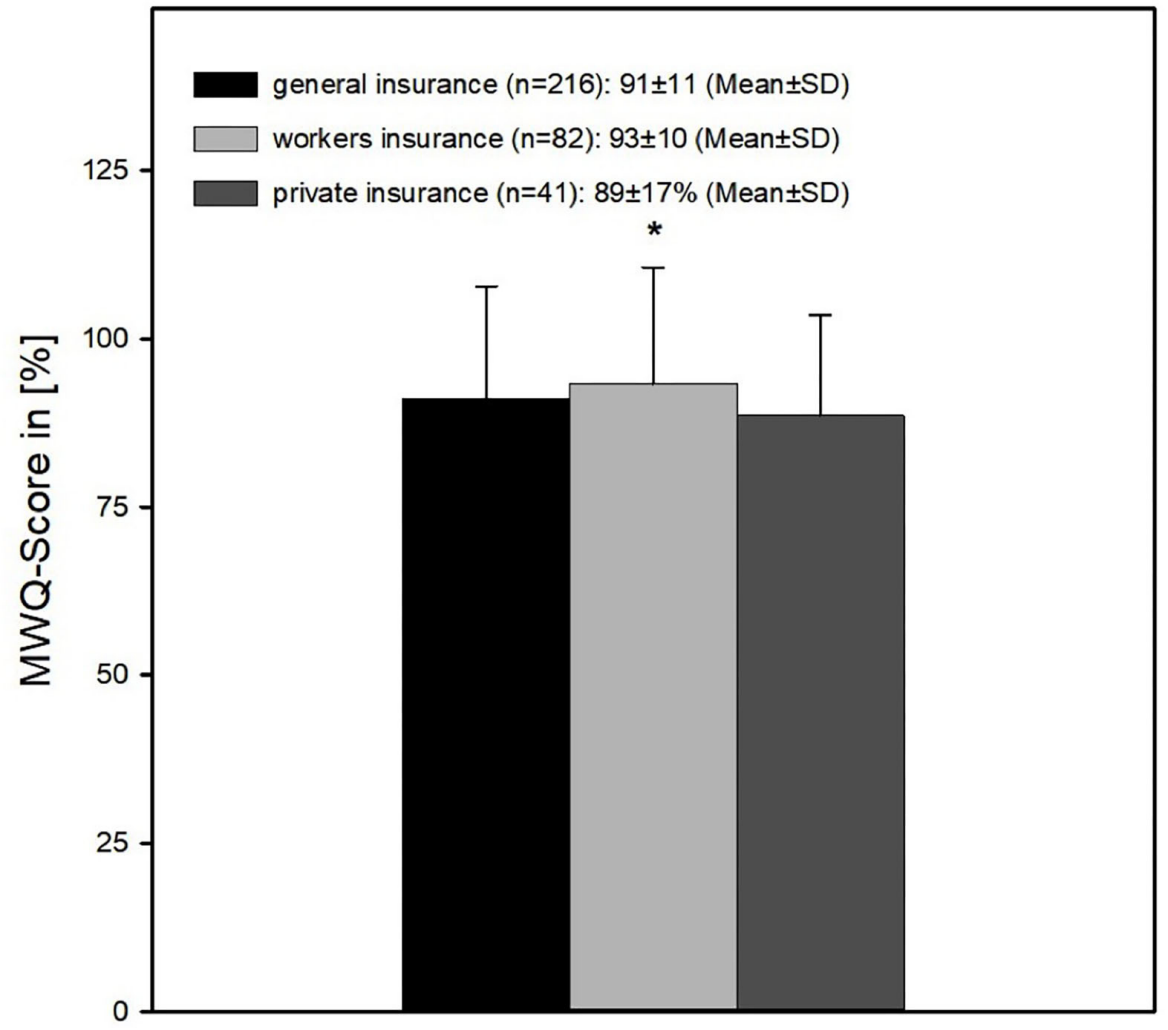
Wrist function in respect of insurance type. The outcome was significantly better of patients with a workers insurance compared to patients with a general insurance (*= 91 ± 11% vs. 93 ± 10%; *p* < 0.05 MWUT). Patients with a private insurance (MWQ: 89 ± 17%) had no significant difference to both other groups.

Patients with a statutory insurance with an MWQ-score of 91 ± 11% had a significant worse outcome than those treated with a trade association insurance (93 ± 10%; *p* < 0.05 MWUT) Patients with a private insurance (MWQ: 89 ± 17%) had no significant difference to both other groups (Figure 9).

There was no correlation between surgery duration and MWQ-Score.

## Discussion

This is the first retrospective register study dealing with distal radius fractures built up with the help of PROM. As PROM we have used the Munich Wrist Questionnaire (MWQ), as it is specific for the wrist function (12). The advantage of MWQ is the measurement of both objective data on wrist function and the measurement of subjective, postoperative patient satisfaction.

The advantages of a retrospective register study created with the help of PROM are numerous. First, data collection is fast, easy and low-budget. With this self-assessment score, a huge amount of data can be achieved from numerous patients. Moreover, the observation period after surgery is quite long. This is one of the advantages when compared to a prospective study.

The drop-out rate is high of course, which might be regarded as one of the disadvantages of the retrospective register study, but the patients enrolled with a complete data set are a representative sample.

Compared to a randomized controlled prospective trial, the election and investigator bias can be excluded (13). When comparing two operation methods with a randomized controlled trial, the surgeon who performs the surgery and who very often examines the patient after surgery is biased. On the one hand, every surgeon has a preference for a surgical method, and on the other hand, every surgeon wants a good result for the patient, which could have an impact on the postoperative examination results. We could exclude this bias in the presented register study.

Our results are representative as they are gender and age matched when regarding the current literature. The enrolled collective exists of 96 male and 243 female patients, which is similar to the collective of Sander et al. (9) (for e.g., female: male 181:87). However, international Studies from Sweden for example (10, 11) included significantly more women than men [e.g., female: male = 388:57 (10)].

Average age of our collective is 57 ± 16 years, which can be compared to the average age from the big German study from Sander et al. (9) (av. age 56.9 years) but is younger than the average in the international literature. In a big Swedish register study of Rundgren et al. the mean age is 62.7 ± 17.6 years, 65.4 ± 16.0 for women and 53.6 ± 20.0 for men. This confirms that men suffering from a distal radius fracture are younger than women. As the male percentage in our popularity is higher than in most published studies (2, 11, 14) the mean age of our patients is lowered.

Compared to the current literature, the complication rate of 7% (25 cases) of our study and the need for revision surgery in 10 cases is rather low. Johnson et al. observed 22 complications in 20 patients with an overall complication rate of 9.7%. Sixteen patients had to undergo revision surgery (15). Thorninger et al. (16) publicated an overall complication rate of 14.6% with a reoperation rate of 10.4%. Moreover, regarding the current literature, a closer look at the type of complications and the mean observation time has to be taken. While most studies have an observation time between 1 (15) and 3.5 to 5 years (16) we had an average observation time of 66 months, 5.5 years. Therefore, late complications like tendon ruptures or late onset carpal tunnel probably won't be recorded by some of the studies (15, 16).

As well-known from the current literature, especially elderly patients, older than 65 years, are susceptible to distal radius fractures (1, 3). Especially women are the ones at risk, which is due to the high rate of osteoporosis in this age (2, 17). These findings are confirmed in the present study. Nevertheless, the outcome after surgical treatment of the distal radius fracture in the elderly patient is desirable. There are no significant differences in results of the MWQ between the patient group older than 65 years and younger than that. These findings come along with the statements from the up-to-date literature, which advocate that surgery for DRF depends on the type of fracture and not on the patient's age (5, 7, 18, 19).

In conclusion, building up a fracture register with the help of PROM has several advantages. A high amount of data can be collected fast, easily and low-budget. However, a certain drop-out rate has to be calculated as not all patients are willing to send the questionnaires back. Nevertheless, PROM is a useful tool not only for an in-house quality control but also for building up a fracture register and collecting date to improve the patients' treatment.

## Data Availability Statement

The raw data supporting the conclusions of this article will be made available by the authors, without undue reservation.

## Ethics Statement

The studies involving human participants were reviewed and approved by Ethics Committee, Klinikum rechts der Isar, Technical University rechts der Isar. The patients/participants provided their written informed consent to participate in this study.

## Author Contributions

JR has substantially contributed to the acquisition and analysis. FM has substantially contributed to the design of the work and has contributed to interpretation of data. PB has substantially contributed to the conception and has revised the work. HA has substantially contributed to the conception, has drafted and revised the work, and has contributed to interpretation of data. All authors read and approved the final manuscript.

## Conflict of Interest

The authors declare that the research was conducted in the absence of any commercial or financial relationships that could be construed as a potential conflict of interest.

## Publisher's Note

All claims expressed in this article are solely those of the authors and do not necessarily represent those of their affiliated organizations, or those of the publisher, the editors and the reviewers. Any product that may be evaluated in this article, or claim that may be made by its manufacturer, is not guaranteed or endorsed by the publisher.
